# Publication of clinical trial protocols – what can we learn?

**DOI:** 10.1186/1757-7241-21-12

**Published:** 2013-03-01

**Authors:** Eirik Skogvoll, Jo Kramer-Johansen

**Affiliations:** 1 Department of Anaesthesia and Intensive Care, St. Olav University Hospital, Trondheim, Norway; 2 Dept. of Anaesthesia, Oslo University Hospital, Oslo, Norway

## 

Scandinavian Journal of Trauma, Resuscitation and Emergency Medicine (SJTREM) receives about 220 submissions for publication every year. Based on the principles of open access publishing, all scientifically sound manuscript that are original and in compliance with ethical and author guidelines will be considered for publication. Such editorial policies are in effect in most journals, and together with rigorous peer-review they are the cornerstones of scientific dissemination. The rejection rates for submitted papers and the citations to published papers are considered when overall quality or impact of a journal is evaluated; however, both these metrics have serious shortcomings and can’t be used for comparison between journals without caution. SJTREM currently rejects 60% of all submitted papers; the current impact factor is 1.85.

In clinical medicine we want to provide our patients with the best possible treatment, and we need to know whether some new intervention is more effective than what we have been used to doing. To find out, we need clinical trials. The crucial point in any comparison is to be fair, and in scientific terms this means to rule out as many sources of bias and confounding as possible. Randomization is a powerful tool to ensure that unknown factors will be evenly distributed among the experimental groups, and the randomized controlled trial (RCT) is the golden standard. But even the most elegant study could end up unpublished if the results are unexpected (or even unwanted!), or the results may be tweaked to satisfy a study sponsor’s financial interests. Fear of such publication bias led to registries of clinical trials. Registration has become required for publication in most medical journals [1], and also regulated by laws in some countries. The purpose of such registration is mainly to have publicly disclosure of the ethical considerations, intentions and rationale before recruiting subjects into the trial, and secondarily it will help avoid planning and funding studies that already are running; finally it may serve as a data repository after the end of the study to provide other researchers opportunity to verify or collate results. For journals that must decide on publication, these registries provide opportunity to verify that the submitted paper adheres to the original plan for conduct and analysis. Even so, a recent analysis of 40 RCTs published after 2005 revealed improper presentation of outcomes or analyses in up to 75% of the studies when compared with the original protocol, and late registration into ClinicalTrials.gov in a similar proportion (url: http://www.ncbi.nlm.nih.gov/books/NBK100613/, accessed January 18, 2013).

The reasons mentioned above give sound reason to publish or make publicly available full protocols of clinical trials. For the editors, the question remains whether the protocol represents a redundant publication when it is already publicly available in registries. For the competitive researcher, the question may be how to get properly rewarded for the huge amount of work that has been put into protocol development, in a system where counting of publications may define your further funding or promotions. An additional issue of grievance for the publisher may be that even if agreeing to publish the protocol, there may be small chances to receive the final results for publication due to an understandable quest for high-impact journals and prestigious papers.

Sten Rubertsson and colleagues here present their protocol from the LINC study, which just finished inclusion of patients. The LINC study is a randomized controlled trial of mechanical chest compressions with LUCAS™ combined with a specific algorithm of chest compressions and defibrillations.

After careful editorial consideration, SJTREM has decided to publish the protocol even if the research and analysis plan have been publicly available on ClinicalTrials.gov since 2008 (NTC 00609778). The published protocol provides additional rationale and background for the trial, as well as a detailed description of the organization of the trial; hopefully it may aid others to organize a clinical trial. However, as the trial is now concluded, there is little left for peer-review to change besides issues of language and clarity. We will therefore print the original protocol more or less as submitted and instead provide a critical commentary of the protocol in this editorial. The authors have the opportunity to respond septely.

As an aid in this process, we will follow the 10-dimensional Trial Quality assessment list suggested by Berger and Alperson [2]. This paper aims at identifying unique aspects that may influence trial quality, and differs from previous systems for trial assessment by proposing that the final “quality score” should be obtained by multiplying individual scores. We have avoided the scoring, though, and apply the list in only in a “check-list” or qualitative way. We will also briefly comment on what was made public on ClinicalTrials.gov, and finally on what we perceive as strengths and weaknesses of the study.

## LINC trial quality

### Randomization

Patients were randomized individually as soon as possible after the decision to treat was made, by opening an envelope containing instructions of whether to use or not use LUCAS. The inclusion criteria are wide, but it is unclear whether the arrest needs to be “witnessed by sight or sound”, as the contrary appears to be a protocol violation. It is not reported neither by what means the randomization table was generated, nor whether the envelopes were tracked (for example by numbering). The latter is necessary for an intention-to-treat analysis and to detect protocol violations. It has been revealed [Sten Rubertsson, personal communications, Resuscitation 2012, Vienna, Austria] that during the early phase of the study ambulance staff were able to foresee the next allocation by carefully inspecting the (slightly transparent) envelopes, necessitating a change to black, opaque envelopes. While the ability to foresee the next treatment allocation may lead to inclusion bias in either direction, the extent is unknown.

To maintain a balance between the study groups at each site, block randomization is a common method. The LINC study employed stratified randomization by EMS site (or perhaps by ambulance station, or even individual ambulances ?) in blocks of six, meaning that each site is guaranteed to have a balanced number of patients in the two arms for each six patient recruited. The block size has allegedly been kept secret to reduce the possibility of knowing the next allocation after five patients have been included. Apparently, however, this has been known to the group of authors.

### Masking

The study is single blinded. By nature of the intervention the allocation is known to the ambulance personnel.

### Allocation concealment

See point 2. It is not described in the protocol whether (or how) treatment allocation is concealed for the personnel who registered the outcome.

### Withdrawals and dropouts

By nature of the study, informed consent cannot be obtained before inclusion. Surviving patients (or next-of-kin acting on their behalf) may withdraw from the study, and they will not be included in what is denoted as the Intention to treat (ITT) population. This may bias the analysis– which by definition is no longer by ITT– but because one does not know which treatment is better it is hard to guess the direction.

### Baseline data

The presenting rhythm has not been recorded, but guessed. It is well known that patients presenting with ventricular fibrillation or ventricular tachycardia (VF/VT) have a better prognosis than patients with pulseless electrical activity (PEA) or asystole (ASY). The chosen design thus precludes inference regarding any differential effects of LUCAS according to presenting rhythm.

### Endpoints

The primary endpoint is 4-hour survival, a surrogate measure. This particular choice is not substantiated. Being alive at 4 hours is an obvious prerequisite for the ultimate goal, which is hospital discharge without severe neurological impairment. The intervention may have become known to the treating physician after admission (see points 2 and 3 above) and 4 hours is not a very long time. It is therefore possible that the decision to withdraw or continue further treatment (e.g. proceed to PCI, induce hypothermia etc.) may have been influenced by knowledge of the experimental intervention. The primary outcome is thus not very robust because of the single-blind nature of the trial.

### Stopping rules

The study was continued after an interim analysis after recruitment of 1500 patients was carried out during spring 2011 by an independent safety committee. Presumably, the strict criterion of p < 0.005 – that either treatment much better than the other – was not met, while a meaningful difference might still be detected.

### Statistical methods

The planned group comparison is via Fishers exact test. It is not reported whether site will be included as random effects, although this may be expected due to the stratified randomization scheme (point 1). The sample size (total 2500 patients) has been calculated assuming a 6% difference for the primary surrogate outcome.

### Measures of variability

Confidence intervals for difference in proportions will be reported. If a random effects model (site) is employed as expected, the variance components as well as intraclass correlation should be reported.

### Multiple testing and adjusting the p-values

Not mentioned.

## What was reported on clinicaltrials.gov?

The website was accessed on 9.th October 2012. With identifier NCT 00609778, the trial was registered first in January 2008 and lastly updated in October 2012. Inclusion was completed in August 2012. Although details are lacking, the presentation is consistent with the present protocol.

## Major strengths and weaknesses of the study

The study has major strengths and the authors should be commended for planning and carrying out relevant clinical research under emergency circumstances. A prospective randomized design is the best way to assess an intervention. The large sample size furthermore allows for detection of a realistic outcome improvement. To recruit the necessary number of patients, national and international collaboration allows for study completion within a realistic time frame. Finally, all patients will be accounted for.

There is however a number of weaknesses with this study; mainly related to generalizability and the potential for bias. As for the ability to generalize the results, the device under investigation is confounded with the CPR algorithm. This precludes inference about the effect of LUCAS itself, so the question of whether mechanic compressions are better than manual compressions during CPR will remain unanswered. Furthermore, the initial rhythm is not recorded, so it is not possible to know whether there is any differential effect here. Regarding potential bias, inclusion into the study may have been influenced by ambulance staff knowing the next allocation (point 1, above). Then the primary outcome may be influenced by knowledge of the intervention (point 6, above). Finally, the proposed analyses are not really by ITT due to the potential effect of selective patient withdrawal (point 4, above).
